# Closed Loop Ultrafiltration Feedback Control in Hemodialysis: A Narrative Review

**DOI:** 10.3390/toxins16080351

**Published:** 2024-08-10

**Authors:** Zijun Dong, Lemuel Rivera Fuentes, Sharon Rao, Peter Kotanko

**Affiliations:** 1 Renal Research Institute, New York, NY 10065, USA; rivera.lem@gmail.com (L.R.F.); srao1@ualberta.ca (S.R.); peter.kotanko@rriny.com (P.K.); 2 Icahn School of Medicine at Mount Sinai, New York, NY 10029, USA

**Keywords:** hemodialysis, ultrafiltration, intradialytic hypotension, blood volume monitoring, relative blood volume, intradialytic morbid events, volume overload

## Abstract

While life-sustaining, hemodialysis is a non-physiological treatment modality that exerts stress on the patient, primarily due to fluid shifts during ultrafiltration. Automated feedback control systems, integrated with sensors that continuously monitor bio-signals such as blood volume, can adjust hemodialysis treatment parameters, e.g., ultrafiltration rate, in real-time. These systems hold promise to mitigate hemodynamic stress, prevent intradialytic hypotension, and improve the removal of water and electrolytes in chronic hemodialysis patients. However, robust evidence supporting their clinical application remains limited. Based on an extensive literature research, we assess feedback-controlled ultrafiltration systems that have emerged over the past three decades in comparison to conventional hemodialysis treatment. We identified 28 clinical studies. Closed loop ultrafiltration control demonstrated effectiveness in 23 of them. No adverse effects of closed loop ultrafiltration control were reported across all trials. Closed loop ultrafiltration control represents an important advancement towards more physiological hemodialysis. Its development is driven by innovations in real-time bio-signals monitoring, advancement in control theory, and artificial intelligence. We expect these innovations will lead to the prevalent adoption of ultrafiltration control in the future, provided its clinical value is substantiated in adequately randomized controlled trials.

## 1. Introduction

The progressively rising age and comorbidity burden in hemodialysis (HD) patients adversely affects their clinical status and treatment tolerance, posing substantial challenges [1]. The intermittent nature and short duration of HD sessions heighten the risk of intradialytic morbid events (IME), including hypotension, nausea, cramps, and arrhythmias. IME can disrupt HD sessions and shorten the effective treatment time, resulting in inadequate fluid removal and solute clearance [2]. While longer and more frequent HD offers potential benefits for hemodynamic stability, its widespread adoption is limited for logistic and economic reasons [3].

One of the primary objectives of HD is to eliminate excess fluid through ultrafiltration (UF) and maintain electrolyte balance to prevent fluid overload and disturbances that may affect multiple organ systems, such as electrical conductivity in the heart. Excessive or insufficient removal of electrolytes such as sodium, potassium, and calcium can lead to significant toxicity, resulting in cardiac arrhythmias, neurological complications, and other life-threatening conditions. Fluid overload is a key driver of cardiovascular morbidity and mortality [4]. Inadequate fluid removal may result in high blood pressure (BP), left ventricular (LV) hypertrophy, pulmonary and intestinal congestions, and inflammation. The UF rate (UFR) is determined by numerous factors, most importantly the level of fluid overload and treatment time. Other aspects are the patient’s weight, BP, overall health, and HD frequency. Intradialytic hypotension (IDH) complicates 10–25% of all HD sessions, depending on the specific definitions of IDH definitions [5]. Rapid UF of excessive fluid can result in a drop in BP and IDH when the UFR exceeds the vascular refilling rate from the interstitial space. Interestingly, intravascular volume reduction may not be the only etiology of IDH, and early-onset IDH is a well-recognized entity [6]. Recurrent episodes of IDH may prevent adequate UF, thus aggravating fluid overload. Volume overload is a highly prevalent complication in end-stage renal disease (ESRD) patients, leading to LV hypertrophy, congestive heart failure, and vascular stiffening [7]. Therefore, IDH impacts patient volume management and, ultimately, cardiovascular outcomes [8].

Ideally, prescriptions of UF volume and rate are based on a sound quantitation of fluid status. The current approaches to clinically assess fluid status and ultrafiltration are recognized as subjective and insufficient. More objective means to quantitate fluid overload include bioimpedance, lung and inferior vena cava ultrasound, and relative blood volume (RBV) measurements. While BP measurement is the cornerstone of hemodynamic monitoring during HD, its intermittent nature with long time intervals between measurements is problematic [9,10]. Near-real-time prediction models have been developed to provide timely warning and measurements to prevent IDH, using machine learning and cloud computing infrastructure [11].

Bioimpedance noninvasively evaluates fluid status and can assist with determining the so-called “dry” weight (i.e., euvolemia) in HD patients by analyzing the electrical properties of tissues. However, it poses shortcomings in predicting IME due to its inability to provide real-time monitoring of interstitial-to-intravascular fluid transients during HD and the absences of uniform standards for different devices [12,13].

Other methods for assessing volume status include lung ultrasound and measurements of the inferior vena cava diameter (IVCD), both of which can be performed at the bedside. Lung ultrasound outperforms clinical signs and symptoms in detecting volume overload, while IVCD correlates with clinical symptoms (e.g., cramps, hypotension, and post-HD fatigue) related to hypovolemia [14]. Nevertheless, drawbacks arise from added staff workload, availability, cost, and the variability of ultrasound results due to its operator-dependent nature.

RBV monitoring exploits the concept that UF increases hematocrit or plasma protein con centration during HD unless balanced by identical rates of plasma refilling from the interstitial fluid compartment [15]. Traditional RBV monitoring, although effective in alleviating IDH symptoms in some studies [16], is passive in nature and relies on healthcare professionals for data interpretation and intervention, such as modification of UF rate. The lack of system autonomy may lead to delayed responses, especially when data are not continuously monitored by the staff.

To address this limitation, RBV-guided feedback-controlled HD (FC-HD) was developed. It allows simultaneous monitoring of patient and treatment parameters (e.g., change in RBV, UFR rate) and real-time adjustment of treatment parameters (e.g., UFR, dialysate sodium). If monitored parameters deviate from the desired range, interventions can be administered, either manually (open loop control) [17] or automatically (closed loop control). The immediate and automated analysis of bio-signals from the patient allows swift responses to seamless adjustments along the predefined treatment trajectory. By avoiding major contractions in BV, FC-HD showed clinical benefits of improved hemodynamic stability in comparison to conventional hemodialysis (CHD) [3,10,18,19,20,21,22,23,24,25,26,27,28,29,30,31,32,33,34,35,36,37,38]. This includes reduced IME incidents [3,10,18,19,20,21,22,23,24,25,26,27,28,29,30,31,33,34,35,36,37,38], stabilized BP [19,23,25,26,28,29,30,32], enhanced dialysis adequacy [21,25,31,32,36], reduction of left ventricular wall dysfunction [27], and improved quality of life [29,32].

This narrative review addresses the status of UF feedback systems and their effectiveness. Through analysis of clinical trials, we seek to understand the effect of FC-HD on IME, volume control, hypotensive episodes, the balance of electrolytes, the removal of uremic toxins, and patients’ quality of life.

## 2. Feedback-Controlled Ultrafiltration: Sensor Technologies

The FC-HD mechanism employs real-time, continuous measurements of RBV as the process variable. RBV monitoring devices measure plasma volume changes that translate into concentration of blood constituents, such as red blood cells (RBC) and the related hematocrit (Hct), or blood protein concentration [39]. These markers can be quantified noninvasively using methods like mass density [40], optical density [41], ultrasound speed [42], viscosity [43], electrical conductivity [44], or Hct [45].

RBV can be calculated when the concentration of constituents changes during HD since only fluid is withdrawn from (or added to, in case of infusions) the plasma water. The change in BV relative to the BV at the start of HD is calculated as follows [15]:

BV change (%) = (IV constituent_start HD_/IV constituent_during HD_ − 1) × 100, (1)

RBV monitoring relies on the assumption that changes of constituent (e.g., Hct) observed in the extracorporeal circuit reflect similar changes in the whole vascular system. This assumption holds if the entire blood volume is a single, well-mixed, and closed compartment. Factors such as hemolysis, blood leaks, transfusions, and exchange between intravascular and interstitial compartment in the case of protein during HD can affect the validity of this assumption. Moreover, the whole-body Hct is lower than that of arterial or venous blood [46] due to dynamic reductions in microvascular Hct in capillaries and venules, known as the Fahraeus effect [47]. Note that, depending on the site of the vascular access, either arterial or venous Hct is measured. In the presence of the intradialytic rise in the ratio of whole-body to arterial or venous Hct, the calculated RBV may underestimate an actual decline in BV [48]. Since the current clinically used RBV systems do not provide information on changes in absolute BV, variability in fluid status before HD contributes to the variability of the RBV course during HD. An identical RBV decrease may be associated with different absolute BV changes in the same patient.

In addition to the filling of the vascular system, hemodynamic stability is influenced by factors like postural changes, exercise, food intake, transfusions, and intravenous fluid administration [49]. These factors may partly explain the varying threshold of RBV reduction at which hypotension occurs between HD sessions in the same individual.

Four commonly used RBV monitoring devices on the market are:

1. Crit-Line (Fresenius Medical Care, Waltham, MA, USA) measures Hct with an optical method. It functions either as a stand-alone device or integrated into the Fresenius 2008T HD machine.
2. Hemoscan (Gambro-Hospal, Medolla, Italy) measures Hb through optical absorbances of monochromatic light. It is incorporated into the Integra HD machine.
3. Nikkiso blood volume monitor (Tokyo, Japan) is available as an add-on for Nikkiso HD machines, where intensity of the reflected light is influenced by RBC and correlates with Hct [50].
4. Fresenius Medical Care blood volume monitor (BVM, Bad Homburg, Germany), integrated into the Fresenius 4008, 5008, and 6008 HD machines, uses an online ultrasound technique that measures the speed of sound in blood, which is dependent on the total protein concentration (plasma proteins and Hb) [51].

## 3. Feedback-Controlled Ultrafiltration in HD: Clinical Results

Through the literature search, we identified 28 clinical trials, 12 of which were randomized controlled trials. The trials examined various outcomes such as IDH, BP, quality of life, urea clearance, and post-dialysis regional wall motion abnormalities. (Table 1) [3,10,18,19,20,21,22,23,24,25,26,27,28,29,30,31,32,33,34,35,36,37,38,52,53,54,55]. We specifically assessed the commercially available feedback devices used in these trials: the Hemocontrol™ biofeedback system (HBS), the BVM, and the Haemo-Master (Nikkiso, Tokyo, Japan).

### 3.1. Hemocontrol

Santoro and colleagues introduced the feedback concept and system, where FC-HD significantly reduced hypotensive episodes (*p* < 0.05) and the need for therapeutic interventions (*p* < 0.05) compared to CHD in five hypotension-prone patients [18]. Building upon this approach, BV tracking, a refined multi-input/multi-output (MIMO) closed loop system, was developed. It included three controlled variables: body weight loss, equivalent conductivity, and BV changes, along with two control variables: UFR and DC [19]. During BV tracking phase, eight hypotension-prone patients showed significantly lower systolic BP changes (*p* < 0.05), fewer IDH episodes (*p* < 0.05), and an overall decrease in complaints. This system was further developed into HBS through collaboration with the Hospal-Gambro research group.

Among the 17 studies evaluating the efficiency of HBS, seven were randomized controlled trials, and three were conducted across multiple sites, with study periods ranging from 4 weeks to 36 months. The first randomized crossover trial demonstrated that HBS was not only associated with fewer hypotensive episodes (HBS: 33% vs. CHD: 81.9%; *p* < 0.001), but also increased dialysis treatment efficacy in hypotension-prone patients, showing a higher equilibrated Kt/V (HBS: 1.12 ± 0.05 vs. CHD: 1.03 ± 0.08; *p* < 0.001) and lower urea rebound (HBS: 6.4 ± 2.3% vs. CHD: 14.2 ± 2.7%; *p* < 0.001) [21]. Furthermore, FC-HD was associated with a remarkable 74% increase in event-free sessions (HBS: 50.8% vs. CHD: 29.2%; *p* < 0.01), steadily improving from 33.3% to 63.9% (*p* < 0.01) [23]. Although the observed benefits were not associated with pre-dialysis BP, post-dialysis BP was significantly higher at the study’s conclusion, suggesting better hemodynamic tolerance and that FC-HD contributes to sustainable hemodynamic stability over time. The characteristics of this extended BV regulation effect may also explain improvements seen in some patients during CHD treatments (a “carryover effect”).

The sustainability of BP stabilization achieved by HBS in hypotension-prone patients was evaluated through continuous monitoring of intra-HD and 24-h post-HD BP [26]. HBS significantly improved intra-HD hemodynamic stability, resulting in higher post-HD systolic BP (*p* < 0.05). However, this effect diminished 16 h after dialysis.

Concerns regarding HBS affecting sodium balance through DC adjustments were addressed in a two-year follow-up study, where subjects’ BP, body weight changes, and serum sodium concentrations remained unchanged, with no adverse effects [22]. HBS, thus, proves effective and safe in reducing IDH morbidity, as evidenced by a significantly lower overall occurrence of symptomatic IDH (HBS: 21.0 ± 0.3% vs. CHD: 31.8 ± 0.4%) and muscle cramps (HBS: 4.8 ± 0.1% vs. CHD: 8.0 ± 0.2%), representing a reduction of 34% and 40%, respectively (*p* < 0.0001).

In IDH-prone patients with DM, HBS significantly reduced muscle cramps by 83.7% and hypotensive episodes by 88.9% [31]. This led to reduced reliance on antihypertensive drugs and positive effects on BP, LV mass index, and cardiac ejection fraction, indicating correction of cardiac issues related to intermittent hyperglycemia. Similar efficacy was observed in a study exclusively involving Asian IDH-prone patients, with similar IDH reduction rate for diabetic (−44.0%) and non-diabetic (−42.4%) patients [36]. The reduced IDH was correlated to post-HD fatigue (r = −0.39, *p* = 0.002) while showing a faster post-HD recovery from fatigue (*p* = 0.048) with the use of HBS. Despite including more diabetic patients and the high prevalence of autonomic dysfunctions in DM, HBS produced results comparable to those of previous studies. As a result, Hyo-Wook et al. suggested that the degree of autonomic neuropathy, rather than DM itself, may contribute to IDH reduction induced by HBS. These findings highlight FC-HD’s potential to improve clinical parameters for HD adequacy and address specific treatment needs of diabetic patients.

In addition to IDH-prone patients, HBS were also effective for those with a history of IDH and minor IME symptoms, resulting in fewer IDH episodes (HBS: 6.3 ± 11.3% vs. CHD: 15.8 ± 18.3%; *p* < 0.05) and minor IME (11.0 ± 12.8% vs. 18.1 ± 16.9%; *p* < 0.05) [24].

Consistent results were observed in non-hypotension prone chronic HD patients, where HBS reduced symptomatic IDH from 3 ± 0.5% to 0.13 ± 0.13% (*p* < 0.001), IDWG from 2.08 ± 0.05 kg to 1.82 ± 0.06 kg (*p* = 0.009), and increased urea removal from 24.9 ± 3 g to 32.7 ± 1.9 g (*p* < 0.01) [25]. As the first authors to demonstrate improved hemodynamic tolerability, reduced IDWG, and enhanced urea clearance in stable patients with HBS, McIntyre et al. suggested potential benefits of FC-HD can be applicable for a broader range of HD patients.

In a randomized trial that did not select participants based on hemodynamic instability or fluid overload, quality of life significantly improved for the HBS group, while the CHD group worsened, highlighting HBS’s capacity to enhance tolerability in long-term HD patients by alleviating the burden of kidney disease [29]. Moreover, the HBS group demonstrated a substantial reduction in sessions requiring IDH-related nursing intervention in contrast to an increase in the CHD group (HBS: −42.9% vs. CHD: +35.7%; *p* = 0.04). These results support findings of reduced saline infusion and nursing interventions in a non-randomized, subject-blinded trial with hypotension-prone patients, where sessions with IDH were reduced by 45% and staff work time by 57%, saving up to 14 work hours during the intervention period [3].

Additionally, HBS was found to be useful in preventing LV regional wall motion abnormalities, based on the observed smaller decreases in stroke volume and cardiac output, as well as a greater increment in pulse rate [27]. This occurrence can serve as a potential target for interventions to improve dialysis related cardiovascular death and cardiac failure.

### 3.2. Blood Volume Monitor

The BVM implements feedback control of UFR to maintain RBV above a predefined threshold, the critical RBV (cRBV), at which a patient is at risk of IDH [51]. The BVM feedback algorithm is available on the 4008, 5008, and 6008 Fresenius Medical Care HD machines and operates in two modes: observational, where RBV is measured and the individual cRBV is determined, and interventional, where feedback control is applied without altering dialysate sodium levels.

Four [32,33,34,38] out of seven trials [28,54,56] have demonstrated improved hemodynamic and intradialytic stability with the use of BVM.

In stable patients who had never received BVM treatments, systolic BP reduction, as an indicator of BP stability, was significantly smaller during the intervention phase, demonstrating a BP-stabilizing effect of BVM (BVM: −3.9 mmHg vs. CHD1: −13.7 CHD2: −11.0 mmHg; *p* = 0.003, and *p* = 0.035, respectively); UF volume was significantly higher (BVM: 2407 mL vs. CHD1: 2266 mL; *p* = 0.035) and dry weight decreased from 73.3 kg to 70.9 kg over the study course (*p* = 0.032) [32].

Similar efficiency is confirmed by three randomized multicenter studies, where IME were reduced by 20% (*p* = 0.02) and 45% *(p* = 0.024) in IDH prone patients and 38% (*p* = 0.022) in volume overload patients [33,34,38].

Although BVM was not able to reduce IME significantly in one non-randomized multicentric study, patients experienced fewer IME during FC-HD (BVM: 0.695 ± 0.547/treatment vs. CHD: 0.785 ± 0.613/treatment; *p* = 0.064) [28]. Furthermore, a significant effect of BVM was observed in a subgroup of 31 (55%) patients with the highest IME rate (IME in at least every second treatment), where IME per treatment decreased by 17.5% (BVM: 0.979 ± 0.543/treatment vs. CHD: 1.185 ± 0.554/treatment; *p* = 0.004). Further research is warranted to identify patient populations that may derive the most benefits from FC-HD.

Concerns were raised considering a recent finding where FC-HD showed no intradialytic stability improvements [54]. In the original protocol, a sample size of 30 patients was targeted to provide 90% statistical power, demonstrating a 30% reduction in IDH (two-sided *p* = 0.05). Despite rescreening, this sample size could not be reached. An additional simulation was conducted after the study’s completion, involving its 24 participants, to achieve an 80% statistical power for the primary outcome. Additionally, BVM requires inputs of the individual cRBV based on previous sessions and needs to be reassessed weekly. Leung et al. did not provide information regarding the variation of cRBV within subjects throughout the study period.

### 3.3. Haemo-Master

Haemo-Master uses optical conductivity to monitor the “differences” of BV (dBV) by transmitting light near the infrared spectrum through the bloodline and measuring the intensity of the reflected light by RBC [38,55]. While cRBV is set manually in BVM, Haemo-Master establishes the ideal dBV curve through the steepness and the calculated dBV final value generated from previous HD sessions for each individual patient. The module continuously measures dRBV and provides options to automatically regulate UFR (BV-UFC) and/or dialysis conductivity (BV-COC) so the patient dRBV follows the ideal curve. Haemo-Master is available on the DBB-05, DBB06, DB-07, DBB-EXA, and DBB-EXA ES Nikkiso HD machines.

In volume overload patients, treatments with BV-COC and BV-UFC activated (UCR) showed no difference to CHD in the rate of complications during the entire intervention phase and when considering sessions where dry weight was reduced (UCR: 39 ± 27% and 47 ± 27% vs. CHD:34 ± 20% and 41 ± 30%) [38]. However, simultaneous feedback control of BV and blood temperature using BVM and BTM showed significantly fewer intradialytic complications than UCR (*p* = 0.028) and CHD (*p* = 0.022).

In the UCR group, 36% of HD sessions were found to have execution mistakes when 112 HD sessions were randomly selected to examine the execution of HD technique, despite training efforts. The principal mistake was having either BV-COC and/or BV-UFC turned off at the treatment start, which occurred in 20 sessions. In eight sessions, the reference line was unavailable, and in six sessions, the reference line was not adapted for the patient to reach the prescribed dry weight, although BP would have allowed this procedure. This extensibility in implementation may have contributed to the absence of significant differences in HD complications.

## 4. Feedback-Controlled Ultrafiltration in HD: Outlook

For nearly three decades, robust evidence supporting the practical application of closed loop biofeedback systems has been limited. Given the observed optimized fluid removal, improved vascular refilling, enhanced cardiovascular outcomes, and reduced IME incidents, it is highly plausible that these systems could positively impact mortality and other hard endpoints. However, this hypothesis remains untested. 

In the only trial that assessed the effect of FC-HD on morbidity, the intervention group was associated with greater non access-related and access-related hospitalizations (adjusted risk ratios of 1.52 and 1.61, respectively; *p* = 0.04 and *p* = 0.01), as well as a higher mortality rate (Crit-Line: 8.7% vs. CHD: 3.3%; *p* = 0.021). A patient monitoring and intervention algorithm was developed to control UFR based on the RBV curves provided by Crit-Line. Unlike previous studies, where BV monitoring is integrated with an automatic feedback system, this open loop control system required manual adjustment of UFR at the bedside and lacked individualized treatment. Furthermore, the monitoring and intervention procedure was not mandated but only encouraged, as Crit-Line was studied as a voluntary adjunct to patient treatment. This highly variable implementation may have occurred within and across dialysis units, potentially leading to the opposing results.

Although various controllers have become available for clinicians and patients to modify parameters potentially central to the multifactorial pathogenesis of IDH, the association between UFR and IDH has become less clear than previously thought [54]. Factors such as bio-incompatibility may trigger IDH episodes occurring in the first half of the HD session [6]. While most studies aim to prevent IDH and improve vascular refilling by maximizing UFR in the first part of the dialysis, a new UF controller system was designed to bring patients’ RBV trajectory into a “favorable” RBV range, that is associated with better patient survival and intact hemodynamic stability during dialysis [56,57]. The controller showed a robust reaction in response to deliberate disruptive interventions (e.g., signal noise; extreme plasma refill rates) in in silico and ex vivo bench experiments. No adverse events were observed in an open loop clinical proof-of-concept study, where the implementation of the UFR suggested by the controller needed approval by a healthcare professional [58]. While initial results are encouraging, it will be important to conduct studies in patients with diverse clinical characteristics to conclusively substantiate the controller’s clinical performance.

Another controller using BP as the primary input to adjust UFR for BP stabilization resulted in fewer and less severe IDH events across 237 treatments for seven hypotension-prone patients [59]. The closed loop module demonstrated an overall 25% reduction in IDH (39% in the most severe episodes) and improved hemodynamic treatment tolerance when transitioning from CHD to FC-HD (*p* = 0.02) in 55 patients from 15 Italian dialysis centers [10].

Additionally, the utilization of artificial intelligence (AI) and machine learning to predict the occurrence of IDH has been steadily growing [11,60]. While there is still a long journey ahead, considering regulatory and ethical aspects and data protection before implementing AI in routine practice, the integration of AI with feedback controlled UF holds high potential. This approach enables simultaneous analysis of vast datasets with multiple variables, while providing individualized interventions in real-time. 

## 5. Conclusions

Designing a more physiological HD is a decades-long quest. Excess fluid accumulation significantly contributes to morbidity and mortality in hemodialysis patients. UF feedback control is one of the approaches that holds potential to reduce fluid overload and its sequelae, such as hypertension, LV hypertrophy, pulmonary congestion, intestinal and peripheral edema, and inflammation. However, overzealous fluid removal may trigger IDH, fluid depletion and reduce residual kidney function, highlighting the importance of a carefully calibrated UF feedback processes. UF feedback control is also a means to reduce IME and improve quality of life.

Modern UF controllers build on novel technologies that enable continuous monitoring of multi-modal bio-signals that serve as input into control systems. FC-HD has demonstrated a notable reduction in the incidence of IDH. While it is conceivable that this reduction translates into long-term improved clinical outcomes, sufficiently powered randomized clinical studies with hard outcomes are warranted to critically examine this claim. Importantly, FC-HD has not shown associations with worsening outcomes, in contrast to previous concerns.

## 6. Materials and Methods

The search was conducted independently by the authors in PubMed, based on the following search terms: (“biofeedback, psychology”[MeSH Terms] OR (“biofeedback”[All Fields] AND “psychology”[All Fields]) OR “psychology biofeedback”[All Fields] OR “biofeedback”[All Fields] OR ((“feedback”[MeSH Terms] OR “feedback”[All Fields] OR “feedbacks”[All Fields] OR “feedback s”[All Fields]) AND (“controlling”[All Fields] OR “controllability”[All Fields] OR “controllable”[All Fields] OR “controllably”[All Fields] OR “controller”[All Fields] OR “controller s”[All Fields] OR “controllers”[All Fields] OR “controlling”[All Fields] OR “controls”[All Fields] OR “prevention and control”[MeSH Subheading] OR (“prevention”[All Fields] AND “control”[All Fields]) OR “prevention and control”[All Fields] OR “control”[All Fields] OR “control groups”[MeSH Terms] OR (“control”[All Fields] AND “groups”[All Fields]) OR “control groups”[All Fields])) OR “Hemocontrol”[All Fields] OR ((“blood volume”[MeSH Terms] OR (“blood”[All Fields] AND “volume”[All Fields]) OR “blood volume”[All Fields]) AND (“track and field”[MeSH Terms] OR (“track”[All Fields] AND “field”[All Fields]) OR “track and field”[All Fields] OR “track”[All Fields] OR “tracks”[All Fields] OR “tracked”[All Fields] OR “tracking”[All Fields] OR “trackings”[All Fields])) OR ((“blood volume”[MeSH Terms] OR (“blood”[All Fields] AND “volume”[All Fields]) OR “blood volume”[All Fields]) AND (“controlling”[All Fields] OR “controllability”[All Fields] OR “controllable”[All Fields] OR “controllably”[All Fields] OR “controller”[All Fields] OR “controller s”[All Fields] OR “controllers”[All Fields] OR “controlling”[All Fields] OR “controls”[All Fields] OR “prevention and control”[MeSH Subheading] OR (“prevention”[All Fields] AND “control”[All Fields]) OR “prevention and control”[All Fields] OR “control”[All Fields] OR “control groups”[MeSH Terms] OR (“control”[All Fields] AND “groups”[All Fields]) OR “control groups”[All Fields])) OR ((“blood volume”[MeSH Terms] OR (“blood”[All Fields] AND “volume”[All Fields]) OR “blood volume”[All Fields]) AND (“monitor”[All Fields] OR “monitor s”[All Fields] OR “monitorable”[All Fields] OR “monitored”[All Fields] OR “monitoring”[All Fields] OR “monitoring s”[All Fields] OR “monitorings”[All Fields] OR “monitorization”[All Fields] OR “monitorize”[All Fields] OR “monitorized”[All Fields] OR “monitors”[All Fields])) OR “BVM”[All Fields] OR “Haemomaster”[All Fields]) AND (“haemodialysis”[All Fields] OR “renal dialysis”[MeSH Terms] OR (“renal”[All Fields] AND “dialysis”[All Fields]) OR “renal dialysis”[All Fields] OR “hemodialysis”[All Fields]).

## Figures and Tables

**Table 1:** Clinical Trials of Feedback Control Systems in Hemodialysis.

Author (Year)	Method				Intervention	Outcomes
	Design ^1^	Study Duration	Sample Size ^2^	Eligibility Criteria		
Non-Randomized Trials						
Santoro et al. (1994) [18]	Prospective, pilot study. Alternating sequential design: [A-B-A]	18 HD sessions	5	>30% IDH frequency	RBV → UFR and DC (HBS prototype) vs. CHD	% BV reduction B: −10.2, A1: −11.2, A2: −11.5; NS IDH (*n*) B: 1, A1: 8, A2: 5; *p* < 0.05
Santoro et al. (1998) [19]	Prospective, pilot study. Single-blind, alternating sequential design: [A-B-A]	12 weeks	8	≥20% IDH frequency	RBV → UFR and DC (HBS) vs. CHD	% BV reduction B: –10.6, A1: −12.3, A2: –12.5; NS % SBP reduction B: –12.4, A1: –20, A2: –17.5; *p* < 0.05 Severe IDH (*n*) B: 3, A1: 26, A2: 16; *p* < 0.05
Basile et al. (2001) [22]	Prospective, multicentric, non-inferior, sequential design. Medium term: [A-B]; short term: [W-A]-[W-B]	Medium term: 20–36 months; Short term: 14 weeks	19	HD ≥ 6 months + ≥20% symptomatic IDH	RBV → UFR and DC (HBS) vs. CHD	Symptomatic IDH Medium term (%): B: 21.0 vs. A: 31.8; *p* < 0.0001 Short term (*n*): B: 26 vs. A: 45; *p* < 0.002 HBS is safe in the medium term.
Begin et al. (2002) [23]	Prospective, alternating sequential design: [BA-BA-BA]	12 weeks	7	≥30% IDH frequency in 3 months	RBV → UFR and DC (HBS) vs. CHD	% Event free session ^3^ B: 50.8 vs. A: 29.2; *p* < 0.01 Post-HD SBP increased over the study course with HBS (*p* = 0.02).
Wolkotte et al. (2002) [24]	Prospective, preliminary, sequential design: [A-B]	9 weeks (20 HD sessions)	16	≥1 IDH or minor IME incidence in 4 weeks ^4^	RBV → UFR and DC (HBS) vs. CHD	% IDH B: 6.3 vs. A: 15.8; *p* < 0.05 % Minor IME B: 11.0 vs. A: 18.1; *p* < 0.05
McIntyre et al. (2003) [25]	Prospective, sequential design: [A-O-B]	8 weeks	15	Chronic HD	RBV → UFR and DC (HBS) vs. CHD	Symptomatic IDH (*n*) B: 0.13 vs. A: 3; *p* < 0.001 % Session with SBP drop > 40% B: 3.5 vs. A: 7.0; *p* < 0.001 Interdialytic weight gain (kg) B: 1.82 vs. A: 2.08; *p* = 0.009 Equilibrated Kt/V B: 1.13 vs. A: 1.01; *p* = 0.01 Urea mass removed (g) B: 32.7 vs. A: 24.9; *p* < 0.01
Franssen et al. (2005) [26]	Prospective, sequential design: [A-B1 (constant weight)-B2 (reduced weight)]	12 weeks	12	HD > 6 months + >50% symptomatic IDH required intervention in 6 weeks	RBV → UFR and DC (HBS) vs. CHD	% IDH required intervention B1: 37, B2: 28, A: 64; *p* < 0.01 0–16-h post-HD BP is higher with HBS (*p* < 0.05). 16–24-h post-HD BP is NS. Post-HD weight reduction is NS.
Garzoni et al. (2007) [28]	Prospective, multicentric, alternating sequential design: [A-B-A-B…]	At least 18 HD sessions	56	HD ≥ 3 months + ≥4 sessions with IME in 4 weeks	RBV → UFR (BVM) vs. CHD	% IME per session All patients (*n* = 51): B: 69.5 vs. A: 78.5; *p* = 0.064 (NS) Patients with the highest IME rate (*n* = 31): B: 97.9 vs. A: 118.5; *p* = 0.004 SBP reduction (mmHg) B: −18.8 vs. A: −22.2; *p* = 0.007 DBP reduction (mmHg) B: −7.8 vs. A: −9.1; *p* = 0.064 Heart rate increase (/min) B: 1.8 vs. A: 2.3; *p* = 0.014
Mancini et al. (2007) [10]	Prospective, multicentric, alternating sequential design: R-[B-A-B-A…]	30 HD sessions	55	≥30% IDH frequency in 2 months	BP→ UFR vs. CHD	% Severe IDH B: 8.3 vs. A: 13.8; *p* = 0.01 Mild IDH is reduced during FC-HD (−12.3%) but NS.
Winkler et al. (2008) [31]	Prospective, cohort, sequential design: R-B	50 weeks	18	IME during HD + DM	RBV → UFR and DC (HBS) vs. baseline	After 48 weeks of HBS 83.7% muscle cramps reduction (*p* < 0.01) 88.9% IDH reduction (*p* < 0.01) 34.8% single-pool Kt/V increase (*p* < 0.05) 33.3% double pool Kt/V increase (*p* < 0.05) 43.4% AntiMed reduction (NS) 4.4% BP reduction (NS) 25.2% LV mass index reduction (*p* < 0.05)
Sentveld et al. (2008) [32]	Prospective, alternating sequential design: [A-O-B-A]	10 weeks	18	Chronic HD + stable cardiac function	RBV → UFR (BVM) vs. CHD	Post-HD SBP (mmHg) B: 143.5 vs. A1: 137.1; *p* = 0.018 B: 143.5 vs. A2: 141.1; *p* = 0.043 SBP reduction (mmHg) B: −3.9 vs. A1: −13.7; *p* = 0.003 B: −3.9 vs. A2: −11.0; *p* = 0.035 Mean UF volume (mL) B: 2407 vs. A1: 2266; *p* = 0.049 Mean dry weight reduced from 73.3 to 70.9 kg (*p* = 0.032). Quality of life ^5^ improved after period B (*p* = 0.035) but inconsistent between phases.
Coli et al. (2011) [35]	Prospective, multicentric, sequential design: [A-B]	7 months	55	≥1 IME or IDH per week in 6 months	Multiple inputs → UFR and DC (proprietary mathematic model)) vs. CHD/HDF	% IDH B: 0.9 vs. A: 58.7; *p* < 0.001 Body weight, IDWG, presession natremia were NS.
Doria et al. (2014) [3]	Prospective, single-blind, sequential design: [A-B]	6 months	10	>20% IDH frequency in 6 months	RBV → UFR and DC (HBS) vs. CHD	Session without IDH (*n*) B: 333 vs. A: 288; *p* < 0.001 Session required intervention (*n*) B: 57 vs. A: 102; *p* < 0.001 % Premature HD termination B: 0.5 vs. A: 3.8; *p* < 0.001 IDH-related staff worktime (min) B: 578 vs. A: 1416; *p* < 0.001
Hyo Wook et al. (2014) [36]	Prospective, multicentric, sequential design: [A-O-B]	18 weeks	60	HD > 3 months + >25% IDH frequency in 1 month	RBV → UFR and DC (HBS) vs. CHD	% Symptomatic IDH B: 38.4 vs. A: 62.1; *p* < 0.001 IDH-related nursing interventions (/session) B: 0.56 vs. A: 0.96; *p* < 0.001 % Post-HD recovery time from fatigue is shorter with HBS (*p* = 0.048)
Ookawara et al. (2020) [54]	Prospective, multicentric, alternating sequential design: R-[A-B-A*]-[B-A-A*]-[A-B-An]-[B-A-A*]-[A-B-An]-[B-A-A*]-[A-B-A*]-[B-A-A*]	12 weeks	48	HD ≥ 3 months + stable cardiac function + a UF-induced BV reduction during HD	RBV → UFR (Haemo-Master) vs. CHD	% IDH prevalence B: 51.6 vs. A: 51.3; NS % Symptomatic IDH intervention B: 4.4 vs. A: 3.9; NS % BV reduction B: −12.1 vs. A: −14.4; *p* < 0.001
Zschätzsch et al. (2021) [55]	Mixed-methods, intra-individual comparison, explorative design: retrospective [A-B1]-prospective [B2]	12 weeks	21	≥4 weeks using Fresenius 5008 with BVM + treated by 5008 without BVM for 4 weeks in 2 years	B1: RBV → UFR (5008 BVM) vs. CHD vs. & B1: RBV → UFR (5008 BVM) vs. B2: RBV → UFR (6008 BVM)	% IME B1: 2.8, B2: 2.5, A: 2.4; NS Kt/V B1: 1.60 vs. A: 1.65; NS B1: 1.55 vs. B2: 1.55; NS Total UF volume (mL) B1: 2344 vs. A: 2189; NS B1: 2316 vs. B2: 2492; *p* = 0.003
Randomized Controlled Trials						
Ronco et al. (2000) [21]	Prospective, crossover, sequential design. Randomization: [AB] or [BA]	4 weeks	12	HD > 6 months + >70% symptomatic IDH in 12 sessions + IDWG > 3 kg + normal hydration status	RBV → UFR and DC (HBS) vs. Acetate-free biofiltration	% IDH B: 33.3 vs. A: 81.9; *p* < 0.001 % Saline infusion B: 20.8 vs. A: 79.2; *p* < 0.001 Single-pool Kt/V B: 1.26 vs. A: 1.34; *p* < 0.005 Equilibrated Kt/V B: 1.12 vs. A: 1.03; *p* < 0.001 % Urea rebound B: 6.4 vs. A: 14.2; *p* < 0.001
Santoro et al. (2002) [20]	Prospective, multicentric, crossover, single-blind, alternating sequential design. Balanced block randomization: R-[ABAB] or R-[BABA]	18 weeks	32	20–80% IDH frequency in 2 months + ≥1 comorbidity (cardiac disease, DM, or hypertension)	RBV → UFR and DC (HBS) vs. CHD	% IDH B: 33.5 vs. A: 23.5; *p* = 0.004 The more IDH in period A, the better response in period B (*p* < 0.001). 10% overall IME reduction in period B (*p* < 0.001).
Reddan et al. (2005) [17]	Prospective, multicentric, open loop algorithm design. Randomization: R-[A] or R-[B]	26 weeks	443	HD ≥ 2 months	RBV → UFR (Crit-Line + intervention algorithm) vs. CHD	Adjusted hospitalization risk ratio (Group B vs. A) non-access related: 1.61; *p* = 0.01 access related: 1.52; *p* = 0.04 % Mortality B: 8.7 vs. A: 3.3; *p* = 0.021
Moret et al. (2006) [52]	Prospective, crossover, sequential design. Block randomization: [A-W-B1-W-B2-W-B3] or [B1-W-B2-W-B3-W-A] or [B2-W-B3-W-A-W-B1] or [B3-W-A-W-B1-W-B2]	4 months	10	>2 incidence of <100 mmHg SBP or a drop > 30 mmHg with IDH symptoms in 3 weeks	B1: Linear sodium profiling vs. B2: RBV → UFR and DC (HBS) vs. B3: Plasma conductivity →DC vs. CHD	% Symptomatic IDH B2: 8 vs. B1: 14 vs. A: 16 vs. B3: 17; NS IDGW and pre-HD SBP are NS.
Selby et al. (2006) [27]	Prospective, crossover, sequential design. Randomization: [O-A-W-B] or [O-B-W-A]	4 weeks	8	IDH prone + >51 g/m LV mass index	RBV → UFR and DC (HBS) vs. CHD	New regional wall motion abnormalities development (*n*) B: 23 vs. A: 42 (odds ratio: 1.8; 95% CI: 1.1–3.0) Asymptomatic IDH (*n*) B: 12 vs. A: 24 (odds ratio: 2.0; 95% CI: 1.01–4.4)
Deziel et al. (2007) [29]	Prospective study. Block randomization (IDH stratified): R-[A] or R-[B]	28 weeks	44	HD ≥ 3 months	RBV → UFR and DC (HBS) vs. CHD	% Session required nursing interventions B: −42.9 vs. A: 35.7; *p* = 0.04 Quality of life change ^6^ B: 5.2 vs. A: −6.2; *p* = 0.004 Overall BP reduction over the study period (*p* = 0.005)
Dasselaar et al. (2007) [30]	Prospective, single-blind design. Block randomization: R-[A] or R-[B]	16 weeks	28	>150/90 mmHg BP in >50% sessions + volume overload + use of antihypertensive drug or cardiothoracic ratio > 0.5	RBV → UFR and DC (HBS) vs. CHD	Pre-HD SBP change (mmHg) B: −22.5; *p* < 0.01 A: 3; NS Pre-HD DBP change (mmHg) B: −8.3; *p* < 0.05 A: 1.2; NS IDH frequency decreased in period B compared to period R (*p* < 0.05).
Nesrallah et al. (2008) [37]	Prospective study. Concealed randomization (DM stratified): R-[A] or R-[B]	28 weeks	60	HD ≥ 6 months + ECV > 45% of total body water	RBV → UFR and DC (HBS) vs. CHD	%ECV change B: 1.8 vs. A: 0.87; NS % IDH per session B: 11 vs. A: 19; *p* = 0.014 BP, use of AntiMed, quality of life ^7^ are NS.
Gabrielli et al. (2009) [33]	Prospective, multicentric, crossover, sequential design. Randomization: R-[A-B] or R-[B-A]	18 weeks	26	≥33% IME Frequency in 6 weeks	RBV → UFR (BVM) vs. CHD	% IME B: 32 vs. A: 40; *p* = 0.02 % Symptomatic IDH B: 24 vs. A: 32; *p* = 0.04 % IME per session B: 42 vs. A: 53; *p* = 0.04 Equilibrated Kt/V B: 1.17 vs. A: 1.12; NS
Veljancic et al. (2011) [34]	Prospective, multicentric, crossover, sequential design. Randomization: R-[A-B] or R-[B-A]	15 weeks	26	Cardiovascular instability history + ≥5 sessions with IME in period R	RBV → UFR (BVM) + BT→ DT (BTM) vs. CHD	% IME B: 18.0 vs. A: 32.8; *p* = 0.024
Antlanger et al. (2017) [38]	Prospective, multicentric design. Block randomization (center stratified): [A] or R-[B1] or R-[B2]	4 weeks	50	HD ≥ 3 months + ≥15% ECV	B1: RBV → UFR and DC (Haemo-Master) vs. B2: RBV → UFR (BVM) + BT → DT (BTM) vs. CHD	% IME B2: 21 vs. A: 34, *p* = 0.022 B2: 21 vs. B1: 39, *p* = 0.028 B1: 39 vs. A: 34; NS Dry weight reduction (%body weight) B2: 5.0 vs. B1: 2.0, *p* = 0.013 B2: 5.0 vs. A: 3.9; NS B1: 2.0 vs. A: 3.9; NS Mean UFR are significantly higher in B2 than in B1 and CHD at similar dialysis times. SBP reduction between groups are NS.
Leung et al. (2017) [53]	Prospective, multicentric, crossover, single-blind, sequential design. Randomization: R-[A-W-B] or R-[B-W-A]	22 weeks	26	HD > 3 months + ≥30% symptomatic IDH in 8 weeks	RBV → UFR (BVM) vs. CHD	Symptomatic IDH per hour B: 0.10 vs. A: 0.07; NS IDWG, brain natriuretic peptide, cardiac troponin, extra-to-intracellular water ratio, and dialysis recovery time are NS

^1^ A represents the study’s control period (A* were excluded in the analysis); B denotes the intervention period; O denotes the optimization period; R represents the run-in period; W denotes the wash out period. ^2^ Number of patients included in the final analysis (completed the study period). ^3^ Event free session indicates sessions that required no therapeutic intervention for hypotension related signs or symptoms. ^4^ Minor IME symptoms were nausea, headache, cramps, or abdominal pain without a decline in BP. ^5^ Quality of life was measured by visual analog scale. ^6^ Quality of life was assessed using the kidney disease and quality of life short form. Higher scores indicate a better quality of life. ^7^ Quality of life was measured using the dialysis somatic symptoms questionnaire. Abbreviations: DC—dialysate conductivity; SBP—systolic BP; DBP—diastolic BP; IDWG—interdialytic weight gain; DM—diabetes mellitus; AntiMed—antihypertensive medication; HDF—hemodiafiltration; CI—confidence interval; ECV—extracellular fluid volume; BT—blood temperature; BTM—blood temperature monitor (Fresenius, Bad Homburg, Germany); DT—dialysate temperature.

## Data Availability

No new data were created in this study. Data sharing is not applicable to this article.

## References

[1] Shoji T., Tsubakihara Y., Fujii M., Imai E. (2004). Hemodialysis-associated hypotension as an independent risk factor for two-year mortality in hemodialysis patients. Kidney Int..

[2] Steuer R.R., Leypoldt J.K., Cheung A.K., Harris D.H., Conis J.M. (1994). Hematocrit as an indicator of blood volume and a predictor of intradialytic morbid events. Asaio J..

[3] Doria M., Genovesi S., Biagi F., Steckiph D., Mancini E., Stella A., Santoro A. (2014). The dialysis staff workload and the blood volume tracking system during the hemodialysis sessions of hypotension-prone patients. Int. J. Artif. Organs.

[4] Dekker M.J., Marcelli D., Canaud B.J., Carioni P., Wang Y., Grassmann A., Konings C.J., Kotanko P., Leunissen K.M., Levin N.W. (2017). Impact of fluid status and inflammation and their interaction on survival: A study in an international hemodialysis patient cohort. Kidney Int..

[5] Sands J.J., Usvyat L.A., Sullivan T., Segal J.H., Zabetakis P., Kotanko P., Maddux F.W., Diaz-Buxo J.A. (2014). Intradialytic hypotension: Frequency, sources of variation and correlation with clinical outcome. Hemodial. Int..

[6] Keane D.F., Raimann J.G., Zhang H., Willetts J., Thijssen S., Kotanko P. (2021). The time of onset of intradialytic hypotension during a hemodialysis session associates with clinical parameters and mortality. Kidney Int..

[7] Thijssen S., Kappel F., Kotanko P. (2013). Absolute blood volume in hemodialysis patients: Why is it relevant, and how to measure it?. Blood Purif..

[8] Daugirdas J.T. (2001). Pathophysiology of dialysis hypotension: An update. Am. J. Kidney Dis..

[9] Assimon M.M., Flythe J.E. (2015). Intradialytic Blood Pressure Abnormalities: The Highs, The Lows and All That Lies Between. Am. J. Nephrol..

[10] Mancini E., Mambelli E., Irpinia M., Gabrielli D., Cascone C., Conte F., Meneghel G., Cavatorta F., Antonelli A., Villa G. (2007). Prevention of dialysis hypotension episodes using fuzzy logic control system. Nephrol. Dial. Transplant..

[11] Zhang H., Wang L.C., Chaudhuri S., Pickering A., Usvyat L., Larkin J., Waguespack P., Kuang Z., Kooman J.P., Maddux F.W. (2023). Real-time prediction of intradialytic hypotension using machine learning and cloud computing infrastructure. Nephrol. Dial. Transplant..

[12] Van der Sande F.M., van de Wal-Visscher E.R., Stuard S., Moissl U., Kooman J.P. (2020). Using Bioimpedance Spectroscopy to Assess Volume Status in Dialysis Patients. Blood Purif..

[13] Dou Y., Zhu F., Kotanko P. (2012). Assessment of extracellular fluid volume and fluid status in hemodialysis patients: Current status and technical advances. Semin. Dial..

[14] Annamalai I., Balasubramaniam S., Fernando M.E., Srinivasaprasad N.D., Suren S., Thirumalvalavan K., Veerakumar A.M. (2019). Volume Assessment in Hemodialysis: A Comparison of Present Methods in Clinical Practice with Sonographic Lung Comets. Indian. J. Nephrol..

[15] Santoro A., Mancini E., Paolini F., Zucchelli P. (1996). Blood volume monitoring and control. Nephrol. Dial. Transplant..

[16] Barth C., Boer W., Garzoni D., Kuenzi T., Ries W., Schaefer R., Schneditz D., Tsobanelis T., van der Sande F., Wojke R. (2003). Characteristics of hypotension-prone haemodialysis patients: Is there a critical relative blood volume?. Nephrol. Dial. Transplant..

[17] Reddan D.N., Szczech L.A., Hasselblad V., Lowrie E.G., Lindsay R.M., Himmelfarb J., Toto R.D., Stivelman J., Winchester J.F., Zillman L.A. (2005). Intradialytic blood volume monitoring in ambulatory hemodialysis patients: A randomized trial. J. Am. Soc. Nephrol..

[18] Santoro A., Mancini E., Paolini F., Spongano M., Zucchelli P. (1994). Automatic control of blood volume trends during hemodialysis. Asaio J..

[19] Santoro A., Mancini E., Paolini F., Cavicchioli G., Bosetto A., Zucchelli P. (1998). Blood volume regulation during hemodialysis. Am. J. Kidney Dis..

[20] Santoro A., Mancini E., Basile C., Amoroso L., Di Giulio S., Usberti M., Colasanti G., Verzetti G., Rocco A., Imbasciati E. (2002). Blood volume controlled hemodialysis in hypotension-prone patients: A randomized, multicenter controlled trial. Kidney Int..

[21] Ronco C., Brendolan A., Milan M., Rodeghiero M.P., Zanella M., La Greca G. (2000). Impact of biofeedback-induced cardiovascular stability on hemodialysis tolerance and efficiency. Kidney Int..

[22] Basile C., Giordano R., Vernaglione L., Montanaro A., De Maio P., De Padova F., Marangi A.L., Di Marco L., Santese D., Semeraro A. (2001). Efficacy and safety of haemodialysis treatment with the Hemocontrol biofeedback system: A prospective medium-term study. Nephrol. Dial. Transplant..

[23] Bégin V., Déziel C., Madore F. (2002). Biofeedback regulation of ultrafiltration and dialysate conductivity for the prevention of hypotension during hemodialysis. Asaio J..

[24] Wolkotte C., Hassell D.R., Moret K., Gerlag P.G., van den Wall Bake A.W., van der Sande F.M., Kooman J.P. (2002). Blood volume control by biofeedback and dialysis-induced symptomatology. A short-term clinical study. Nephron.

[25] McIntyre C.W., Lambie S.H., Fluck R.J. (2003). Biofeedback controlled hemodialysis (BF-HD) reduces symptoms and increases both hemodynamic tolerability and dialysis adequacy in non-hypotension prone stable patients. Clin. Nephrol..

[26] Franssen C.F., Dasselaar J.J., Sytsma P., Burgerhof J.G., de Jong P.E., Huisman R.M. (2005). Automatic feedback control of relative blood volume changes during hemodialysis improves blood pressure stability during and after dialysis. Hemodial. Int..

[27] Selby N.M., Lambie S.H., Camici P.G., Baker C.S., McIntyre C.W. (2006). Occurrence of regional left ventricular dysfunction in patients undergoing standard and biofeedback dialysis. Am. J. Kidney Dis..

[28] Garzoni D., Keusch G., Kleinoeder T., Martin H., Dhondt A., Cremaschi L., Tatsis E., Ibrahim N., Boer W., Kuehne S. (2007). Reduced complications during hemodialysis by automatic blood volume controlled ultrafiltration. Int. J. Artif. Organs.

[29] Déziel C., Bouchard J., Zellweger M., Madore F. (2007). Impact of hemocontrol on hypertension, nursing interventions, and quality of life: A randomized, controlled trial. Clin. J. Am. Soc. Nephrol..

[30] Dasselaar J.J., Huisman R.M., de Jong P.E., Burgerhof J.G., Franssen C.F. (2007). Effects of relative blood volume-controlled hemodialysis on blood pressure and volume status in hypertensive patients. Asaio J..

[31] Winkler R.E., Pätow W., Ahrenholz P. (2008). Blood volume monitoring. Contrib. Nephrol..

[32] Sentveld B., van den Brink M., Brulez H.F., Potter van Loon B.J., Weijmer M.C., Siegert C.E. (2008). The influence of blood volume-controlled ultrafiltration on hemodynamic stability and quality of life. Hemodial. Int..

[33] Gabrielli D., Krystal B., Katzarski K., Youssef M., Hachache T., Lopot F., Lasseur C., Gunne T., Draganov B., Wojke R. (2009). Improved intradialytic stability during haemodialysis with blood volume-controlled ultrafiltration. J. Nephrol..

[34] Veljančic L., Popović J., Radović M., Ahrenholz P., Ries W., Frenken L., Wojke R. (2011). Simultaneous blood temperature control and blood volume control reduces intradialytic symptoms. Int. J. Artif. Organs.

[35] Colì L., La Manna G., Comai G., Ursino M., Ricci D., Piccari M., Locatelli F., Di Filippo S., Cristinelli L., Bacchi M. (2011). Automatic adaptive system dialysis for hemodialysis-associated hypotension and intolerance: A noncontrolled multicenter trial. Am. J. Kidney Dis..

[36] Gil H.W., Bang K., Lee S.Y., Han B.G., Kim J.K., Kim Y.O., Song H.C., Kwon Y.J., Kim Y.S. (2014). Efficacy of hemocontrol biofeedback system in intradialytic hypotension-prone hemodialysis patients. J. Korean Med. Sci..

[37] Nesrallah G.E., Suri R.S., Thiessen-Philbrook H., Heidenheim P., Lindsay R.M. (2008). Can extracellular fluid volume expansion in hemodialysis patients be safely reduced using the hemocontrol biofeedback algorithm? A randomized trial. Asaio J..

[38] Antlanger M., Josten P., Kammer M., Exner I., Lorenz-Turnheim K., Eigner M., Paul G., Klauser-Braun R., Sunder-Plassmann G., Säemann M.D. (2017). Blood volume-monitored regulation of ultrafiltration to decrease the dry weight in fluid-overloaded hemodialysis patients: A randomized controlled trial. BMC Nephrol..

[39] Schneditz D., Pogglitsch H., Horina J., Binswanger U. (1990). A blood protein monitor for the continuous measurement of blood volume changes during hemodialysis. Kidney Int..

[40] Kenner T., Hinghofer-Szalkay H., Leopold H., Pogglitsch H. (1977). [The relation between the density of blood and the arterial pressure in animal experiments and in patients during hemodialysis (author’s transl)]. Z. Kardiol..

[41] Wilkinson J.S., Fleming S.J., Greenwood R.N., Cattell W.R., Aldridge C. (1987). Continuous measurement of blood hydration during ultrafiltration using optical methods. Med. Biol. Eng. Comput..

[42] Schneditz D., Kenner T., Heimel H.D., Stabinger H. (1989). A sound-speed sensor for the measurement of total protein concentration in disposable, blood-perfused tubes. J. Acoust. Soc. Am..

[43] Greenwood R.N., Aldridge C., Cattell W.R. (1984). Serial blood water estimations and in-line blood viscometry: The continuous measurement of blood volume during dialysis procedures. Clin. Sci..

[44] De Vries P.M., Kouw P.M., Meijer J.H., Oe L.P., Schneider H., Donker A.J. (1988). Changes in blood parameters during hemodialysis as determined by conductivity measurements. ASAIO Trans..

[45] Schallenberg U., Stiller S., Mann H. (1987). A new method of continuous haemoglobinometric measurement of blood volume during haemodialysis. Life Support. Syst..

[46] Harrison M.H., Graveney M.J., Cochrane L.A. (1982). Some sources of error in the calculation of relative change in plasma volume. Eur. J. Appl. Physiol. Occup. Physiol..

[47] Goldsmith H.L., Cokelet G.R., Gaehtgens P. (1989). Robin Fåhraeus: Evolution of his concepts in cardiovascular physiology. Am. J. Physiol..

[48] Mitra S., Chamney P., Greenwood R., Farrington K. (2004). The relationship between systemic and whole-body hematocrit is not constant during ultrafiltration on hemodialysis. J. Am. Soc. Nephrol..

[49] Franssen C., Dasselaar J., Huisman R. (2004). Characteristics of hypotension-prone haemodialysis patients: Is there a critical relative blood volume?. Nephrol. Dial. Transplant..

[50] Yoshida I., Ando K., Ando Y., Ookawara S., Suzuki M., Furuya H., Iimura O., Takada D., Kajiya M., Komada T. (2010). A new device to monitor blood volume in hemodialysis patients. Ther. Apher. Dial..

[51] Schneditz D., Moser M., Smolle-Jüttner F.M., Dörp E., Pogglitsch H., Kenner T. (1990). Methods in clinical hemorheology: The continuous measurement of arterial blood density and blood sound speed in man. Biorheology.

[52] Moret K., Aalten J., van den Wall Bake W., Gerlag P.G.G., Beerenhout C.M., van der Sande F.M., Leunissen K.M.L., Kooman J.P. (2006). The effect of sodium profiling and feedback technologies on plasma conductivity and ionic mass balance: A study in hypotension-prone dialysis patients. Nephrol. Dial. Transplant. Off. Publ. Eur. Dial. Transplant. Assoc.-Eur. Ren. Assoc..

[53] Leung K.C.W., Quinn R.R., Ravani P., Duff H., MacRae J.M. (2017). Randomized Crossover Trial of Blood Volume Monitoring-Guided Ultrafiltration Biofeedback to Reduce Intradialytic Hypotensive Episodes with Hemodialysis. Clin. J. Am. Soc. Nephrol..

[54] Ookawara S., Ito K., Uchida T., Tokuyama K., Kiryu S., Suganuma T., Hojyo K., Miyazawa H., Ueda Y., Ito C. (2020). Hemodialysis crossover study using a relative blood volume change-guided ultrafiltration control compared with standard hemodialysis: The BV-UFC study. Ren. Replace. Ther..

[55] Zschätzsch S., Stauss-Grabo M., Gauly A., Braun J. (2021). Integrating Monitoring of Volume Status and Blood Volume-Controlled Ultrafiltration into Extracorporeal Kidney Replacement Therapy. Int. J. Nephrol. Renovasc Dis..

[56] Preciado P., Zhang H., Thijssen S., Kooman J.P., van der Sande F.M., Kotanko P. (2019). All-cause mortality in relation to changes in relative blood volume during hemodialysis. Nephrol. Dial. Transplant..

[57] Casper S., Fuertinger D.H., Tapia Silva L.M., Rivera Fuentes L., Thijssen S., Kotanko P. (2022). Proportional integral feedback control of ultrafiltration rate in hemodialysis. Int. J. Artif. Organs.

[58] Rivera-Fuentes L., Tapia L., Thijssen S., Rogg S., Kotanko P. (2020). P1079NOVEL ultrafiltration rate feedback controller for attainment of relative blood volume targets during hemodialysis. Nephrol. Dial. Transplant..

[59] Schmidt R., Roeher O., Hickstein H., Korth S. (2001). Prevention of haemodialysis-induced hypotension by biofeedback control of ultrafiltration and infusion. Nephrol. Dial. Transplant..

[60] Randhay A., Eldehni M.T., Selby N.M. (2023). Feedback control in hemodialysis. Semin. Dial..

